# Supplementary material for: Human hippocampal CA3 damage disrupts both recent and remote episodic memories
Source: eLife. 2020 Jan 24;9:e41836. doi: 10.7554/eLife.41836 (PMC6980860; doi:10.7554/eLife.41836)
Supplement: Supplementary file 1. — (a) Neuropsychological domain performance in the amnesic group. (b) Results from graph theoretic analyses of the default network (DN). Between-group differences in global efficiency, local efficiency, betweenness centrality, average path length, clustering coefficient, and degree were examined. Network edges (adjacency matrix threshold) were thresholded at a z-score > 0.84 (one-sided, positive) and assessed at a corrected analysis threshold (p-FDR <0.05, two-sided) and, for completeness, at an uncorrected analysis threshold (p-uncorrected <0.05, two-sided). (c) Results from graph theoretic analyses of the somatomotor network. Between-group differences in global efficiency, local efficiency, betweenness centrality, average path length, clustering coefficient, and degree were examined. Network edges (adjacency matrix threshold) were thresholded at a z-score > 0.84 (one-sided, positive) and assessed at a corrected analysis threshold (p-FDR <0.05, two-sided), and, for completeness, at an uncorrected analysis threshold (p-uncorrected <0.05, two-sided). (d) Results from graph theoretic analyses of the visual network. Between-group differences in global efficiency, local efficiency, betweenness centrality, average path length, clustering coefficient, and degree were examined. Network edges (adjacency matrix threshold) were thresholded at a z-score > 0.84 (one-sided, positive) and assessed at a corrected (p-FDR <0.05, two-sided), and, for completeness, at an uncorrected analysis threshold (p-uncorrected <0.05, two-sided). (e) Results from graph theoretic analyses of the salience network. Between-group differences in global efficiency, local efficiency, betweenness centrality, average path length, clustering coefficient, and degree were examined. Network edges (adjacency matrix threshold) were thresholded at a z-score > 0.84 (one-sided, positive) and assessed at a corrected (p-FDR <0.05, two-sided), and, for completeness, at an uncorrected analysis threshold (p-uncorrected < [file elife-41836-supp1.docx]

**Supplementary Tables**

**Neuropsychological assessment**

**Supplementary file 1a.** **Neuropsychological domain performance in the amnesic group**

| Neuropsychological | n. | ave. *z*-score | s.e.m. | *t* | *d.f.* | p-value |
| --- | --- | --- | --- | --- | --- | --- |
| Domain |  |  |  |  |  |  |
| Intelligence | 15 | 0.79 | 0.22 | 3.57 | 14 | <0.003* |
| Verbal memory | 16 | -0.42 | 0.22 | -1.91 | 15 | 0.075 |
| Visual memory | 16 | -0.29 | 0.16 | -1.81 | 15 | 0.090 |
| Recognition Memory | 16 | -0.23 | 0.22 | -1.04 | 15 | 0.313 |
| Attention | 15 | 0.12 | 0.15 | 0.80 | 14 | 0.436 |
| Executive Function | 16 | 0.55 | 0.17 | 3.16 | 15 | 0.006* |
| Language | 16 | 0.41 | 0.26 | 1.59 | 15 | 0.134 |
| Visuomotor skills | 16 | 0.09 | 0.09 | 0.99 | 15 | 0.339 |
| Visuoconstructive skills | 16 | -0.03 | 0.34 | -0.10 | 15 | 0.924 |
|  |  |  |  |  |  |  |

Domain scores are based on neuropsychological subtests that are described within the Methods section. *Patient group statistically different from normative data, p<0.05, two-tailed one-sample *t*-test; n.=number of patients tested; ave. *z*-score=average *z*-score; s.e.m.=standard error of the mean; d.f., degrees of freedom. Notably, delayed verbal recall performance (which contributed to the verbal memory domain) was significantly different from normative data (n = 16, ave. *z*-score = -0.77, s.e.m. = 0.24, *t*_(15)_ = -3.16, p = 0.006). Delayed verbal recall was comprised of Logical Memory II, Logical Memory II themes and Word Lists II (WMS-III) and People Recall Test, whereas delayed visual recall (comprised of Rey Delayed Recall) was intact (ave. *z* = -0.08, s.e.m. = 0.20, *t*_(15)_ = 0.41, p = 0.685).

**Graph theoretic analyses**

**Supplementary file 1b. Results from graph theoretic analyses of the default network (DN)**. Between-group differences in global efficiency, local efficiency, betweenness centrality, average path length, clustering coefficient, and degree were examined. Network edges (adjacency matrix threshold) were thresholded at a *z*-score > 0.84 (one-sided, positive) and assessed at a corrected analysis threshold (*p*-FDR <0.05, two-sided), and, for completeness, at an uncorrected analysis threshold (*p*-uncorrected <0.05, two-sided).

| Analysis measure | ROI | *β* | *t* | p-unc. | p-FDR |
| --- | --- | --- | --- | --- | --- |
|  |  |  |  |  |  |
|  |  |  |  |  |  |
| Global Efficiency | network | -0.03 | -1.09 | 0.287 |  |
|  |  |  |  |  |  |
| Local Efficiency | network | 0.13 | 2.33 | 0.027 |  |
|  |  |  |  |  |  |
|  | Left PHC | 0.45 | 3.25 | 0.006 | *0.037 |
|  | Left Rsp | 0.30 | 2.26 | 0.033 | 0.165 |
|  |  |  |  |  |  |
|  | Right Rsp | 0.36 | 3.45 | 0.002 | *0.020 |
|  |  |  |  |  |  |
|  | Left PCC | 0.35 | 3.49 | 0.002 | *0.020 |
| Betweenness Centrality | network | -0.03 | -2.50 | 0.019 |  |
|  | Left PCC | -0.14 | -2.45 | 0.021 | 0.416 |
| Average Path Length | network | -0.66 | -2.91 | 0.007 |  |
|  |  |  |  |  |  |
|  | Left PHC | -1.41 | -3.51 | 0.002 | *0.013 |
|  |  |  |  |  |  |
|  | Right PHC | -1.40 | -3.53 | 0.002 | *0.013 |
|  |  |  |  |  |  |
|  | Left HF | -0.93 | -2.72 | 0.012 | *0.033 |
|  |  |  |  |  |  |
|  | Right HF | -1.18 | -2.99 | 0.006 | *0.020 |
|  |  |  |  |  |  |
|  | Left Rsp | -0.75 | -3.55 | 0.002 | *0.013 |
|  |  |  |  |  |  |
|  | vmPFC | -1.52 | -3.32 | 0.005 | *0.020 |
|  |  |  |  |  |  |
|  | Left TempP | -1.18 | -3.34 | 0.003 | *0.013 |
|  |  |  |  |  |  |
|  | Right PCC | -0.61 | -2.39 | 0.024 | 0.059 |
|  | Left PCC | -0.50 | -2.11 | 0.044 | 0.098 |
| Clustering Coefficient | network | 0.11 | 2.05 | 0.050 |  |
|  |  |  |  |  |  |
|  | Left PHC | 0.31 | 2.24 | 0.042 | 0.254 |
|  |  |  |  |  |  |
|  | Left PCC | 0.23 | 2.42 | 0.024 | 0.235 |
|  |  |  |  |  |  |
|  | Right Rsp | 0.33 | 2..05 | 0.003 | 0.065 |
|  |  |  |  |  |  |
| Degree | network | 0.27 | 1.80 | 0.084 |  |
|  |  |  |  |  |  |

ROI = region-of-interest; *β* = beta value, *t* = *t*-statistic; *d.f*. = degrees of freedom; p-unc. = p-uncorrected at <0.05; p-FDR = p-False Discovery Rate corrected at <0.05; “network” = statistical test comparing entire network across two groups. * significant at p-FDR <0.05.

**Network topology of somatomotor, salience, ventral attention, dorsal attention, and visual resting-state networks were not different between the amnesic and control groups**

Analyses with nodes and edges that corresponded to five other large-scale RSNs previously associated, in other studies, with alterations in functional connectivity following hippocampal and MTL damage were conducted to assess the scalar extent of altered topology in the amnesic group relative to the control group. The RSNs were somatomotor network, salience network, ventral attention network, dorsal attention networks, and the visual network. Co-ordinates for these networks were derived from 13-module parcellation of the 264-node groundtruth graph reported by Power et al. (2011) (**Supplementary file 1n**). These networks were assessed using the same *a priori* graph theoretic metrics and thresholding criteria as those used to test between-group differences in the DN. None of these networks exhibited significant altered topological properties on any of the graph theoretic metrics in a contrast between the amnesic and control group participants (**Supplementary files 1c-1g**).

**Supplementary file 1c. Results from graph theoretic analyses of the somatomotor network**. Between-group differences in global efficiency, local efficiency, betweenness centrality, average path length, clustering coefficient, and degree were examined. Network edges (adjacency matrix threshold) were thresholded at a *z*-score > 0.84 (one-sided, positive) and assessed at a corrected threshold (p-FDR<0.05, two-sided), and, for completeness, at an uncorrected analysis threshold (p-uncorrected <0.05, two-sided).

| Analysis measure | MNI  co-ordinates (x,y,z) | *β* | *t* | p-unc. | p-FDR |
| --- | --- | --- | --- | --- | --- |
|  |  |  |  |  |  |
| Global Efficiency | network | -0.02 | -1.34 | 0.190 |  |
|  |  |  |  |  |  |
|  | -7, -21, 65 | -0.07 | -3.39 | 0.062 | 0.062 |
|  |  |  |  |  |  |
|  | -21, -31, 61 | -0.07 | -2.50 | 0.019 | 0.271 |
|  |  |  |  |  |  |
|  | -23, -30, 72 | -0.07 | -2.08 | 0.047 | 0.454 |
|  |  |  |  |  |  |
| Local Efficiency | network | -0.01 | -0.37 | 0.716 |  |
|  | 0, -15, 47 | 0.21 | 2.09 | 0.047 | 0.572 |
| Betweenness Centrality | network | -0.00 | -0.78 | 0.442 |  |
|  | -7, -33, 72 | 0.04 | 2.07 | 0.048 | 0.971 |
| Average Path Length | network | -0.11 | -0.74 | 0.466 |  |
|  |  |  |  |  |  |
|  | 44, -8, 57 | -0.77 | -2.25 | 0.036 | 0.848 |
|  |  |  |  |  |  |
|  | Left PCC | 0.23 | 2.42 | 0.024 | 0.235 |
|  |  |  |  |  |  |
|  | Left PHC | 0.31 | 2.24 | 0.042 | 0.254 |
|  |  |  |  |  |  |
| Clustering Coefficient | network | -0.01 | -0.40 | 0.696 |  |
|  |  |  |  |  |  |
| Degree | network | -0 | -0.97 | 0.341 |  |
|  |  |  |  |  |  |
|  | -7, -21, 65 | -2.21 | -2.22 | 0.035 | 0.377 |
|  |  |  |  |  |  |
|  | 47, -30, 49 | 1.87 | 2.22 | 0.035 | 0.377 |
|  |  |  |  |  |  |

MNI = Montreal Neurological Institute; *β* = beta value; *t* = *t*-statistic; *d.f.* = degrees of freedom; p-unc. = p-uncorrected at <0.05; p-FDR = p-False Discovery Rate set at <0.05; and, “network” = statistical test comparing entire network across two groups.

**Supplementary file 1d. Results from graph theoretic analyses of the visual network**. Between-group differences in global efficiency, local efficiency, betweenness centrality, average path length, clustering coefficient, and degree were examined. Network edges (adjacency matrix threshold) were thresholded at a *z*-score > 0.84 (one-sided, positive) and assessed at a corrected analysis threshold (p-FDR <0.05, two-sided), and, for completeness, at an uncorrected analysis threshold (p-uncorrected <0.05, two-sided).

| Analysis measure | MNI  co-ordinates  (x,y,z) | *β* | *t* | p-unc. | p-FDR |
| --- | --- | --- | --- | --- | --- |
|  |  |  |  |  |  |
| Global Efficiency | network | -0.01 | -0.54 | 0.593 |  |
|  |  |  |  |  |  |
| Local Efficiency | network | 0.05 | 2.01 | 0.054 |  |
| Betweenness Centrality | network | -0.00 | -044 | 0.667 |  |
|  | 43, -78, -12 | -0.03 | -2.24 | 0.034 | 0.962 |
| Average Path Length | network | -0.04 | -0.34 | 0.736 |  |
|  |  |  |  |  |  |
| Clustering Coefficient | network | 0.05 | 1.86 | 0.073 |  |
|  |  |  |  |  |  |
| Degree | network | 0.07 | 0.052 | 0.606 |  |
|  |  |  |  |  |  |

MNI = Montreal Neurological Institute; *β* = beta value; *t* = *t*-statistic; *d.f.* = degrees of freedom; p-unc. = p-uncorrected at <0.05; p-FDR = p-False Discovery Rate set at <0.05; and, “network” = statistical test comparing entire network across two groups.

**Supplementary file 1e. Results from graph theoretic analyses of the salience network**. Between-group differences in global efficiency, local efficiency, betweenness centrality, average path length, clustering coefficient, and degree were examined. Network edges (adjacency matrix threshold) were thresholded at a *z*-score > 0.84 (one-sided, positive) and assessed at a corrected analysis threshold (p-FDR <0.05, two-sided), and, for completeness, an uncorrected analysis threshold (p-uncorrected <0.05, two-sided).

| Analysis measure | MNI  co-ordinates  (x,y,z) | *β* | *t* | p-unc. | p-FDR |
| --- | --- | --- | --- | --- | --- |
|  |  |  |  |  |  |
| Global Efficiency | network | 0.02 | 0.74 | 0.463 |  |
|  |  |  |  |  |  |
| Local Efficiency | network | -0.04 | -1.23 | 0.230 |  |
|  |  |  |  |  |  |
|  | -28, 52, 21 | 0.73 | 6.20 | 0.003 | 0.062 |
|  |  |  |  |  |  |
|  | 55, -45, 37 | -0.33 | -2.28 | 0.033 | 0.301 |
| Betweenness Centrality | network | 0.01 | -0.54 | 0.591 |  |
| Average Path Length | network | 0.12 | 0.50 | 0.620 |  |
|  |  |  |  |  |  |
|  | -39, 51, 17 | 0.73 | 2.34 | 0.028 | 0.414 |
|  |  |  |  |  |  |
|  | 31, 56, 14 | 0.64 | 2.11 | 0.046 | 0.414 |
|  |  |  |  |  |  |
| Clustering Coefficient | network | -0.05 | -1.29 | 0.206 |  |
|  |  |  |  |  |  |
|  | -28, 52, 21 | -0.31 | -2,55 | 0.024 | 0.269 |
|  |  |  |  |  |  |
|  | 31, 33, 26 | -0.31 | -2.33 | 0.030 | 0.269 |
|  |  |  |  |  |  |
|  | 55, -45, 37 | 0.57 | 2.84 | 0.047 | 0.282 |
|  |  |  |  |  |  |
| Degree | network | -0.00 | -0.02 | 0.987 |  |
|  |  |  |  |  |  |
|  | 37, 32, -2 | -1.25 | -3.26 | 0.003 | 0.054 |
|  |  |  |  |  |  |

MNI = Montreal Neurological Institute; *β* = beta value; *t* = *t*-statistic; *d.f.* = degrees of freedom; p-unc. = p-uncorrected at <0.05; p-FDR = p-False Discovery Rate set at <0.05; and, “network” = statistical test comparing entire network across two groups.

**Supplementary file 1f. Results from graph theoretic analyses of the ventral attention network**. Between-group differences in global efficiency, local efficiency, betweenness centrality, average path length, clustering coefficient and degree were examined. Network edges (adjacency matrix threshold) were thresholded at a *z*-score > 0.84 (one-sided, positive) and assessed at a corrected analysis threshold (p-FDR <0.05, two-sided), and, for completeness, at an uncorrected analysis threshold (p-uncorrected <0.05, two-sided).

| Analysis measure | MNI  co-ordinates  (x,y,z) | *β* | *t* | p-unc. | p-FDR |
| --- | --- | --- | --- | --- | --- |
|  |  |  |  |  |  |
| Global Efficiency | network | -0.07 | -2.59 | 0.0.015 |  |
|  |  |  |  |  |  |
|  | -49, 21 ,-1 | -0.10 | -2.62 | 0.014 | 0.095 |
|  |  |  |  |  |  |
|  | -56, -50, 10 | -0.16 | -2.39 | 0.024 | 0.095 |
|  |  |  |  |  |  |
|  | 53, 33, 1 | -0.08 | -2.31 | 0.028 | 0.095 |
|  |  |  |  |  |  |
|  | 56, -46, 11 | -0.14 | -2.05 | 0.050 | 0.123 |
|  |  |  |  |  |  |
| Local Efficiency | network | 0.06 | 0.58 | 0.568 |  |
| Betweenness Centrality | network | -0.03 | -1.48 | 0.149 |  |
|  | -49, 25, 1 | -0.05 | -2.40 | 0.023 | 0.233 |
| Average Path Length | network | -0.16 | -1.01 | 0.323 |  |
|  |  |  |  |  |  |
| Clustering Coefficient | network | 0.08 | 0.73 | 0.473 |  |
|  |  |  |  |  |  |
| Degree | network | -0.27 | -2.45 | 0.021 |  |
|  |  |  |  |  |  |
|  | -49, 25, -1 | -1.00 | -2.64 | 0.014 | 0.135 |
|  |  |  |  |  |  |

MNI = Montreal Neurological Institute; *β* = beta value; *t* = *t*-statistic; *d.f.* = degrees of freedom; p-unc. = p-uncorrected at <0.05; p-FDR = p-False Discovery Rate set at <0.05; and, “network” = statistical test comparing entire network across two groups.

**Supplementary file 1g. Results from graph theoretic analyses of the dorsal attention network**. Between-group differences in global efficiency, local efficiency, betweenness centrality, average path length, clustering coefficient, and degree were examined. Network edges (adjacency matrix threshold) were thresholded at a *z*-score > 0.84 (one-sided, positive) and assessed at a corrected analysis threshold (p-FDR <0.05, two-sided), and, for completeness, at an uncorrected analysis threshold (p-uncorrected <0.05, two-sided).

| Analysis measure | MNI  co-ordinates  (x,y,z) | *β* | *t* | p-unc. | p-FDR |
| --- | --- | --- | --- | --- | --- |
|  |  |  |  |  |  |
|  |  |  |  |  |  |
| Global Efficiency | network | -0.02 | -0.67 | 0.510 |  |
|  |  |  |  |  |  |
| Local Efficiency | network | -0.10 | -1.25 | 0.221 |  |
|  |  |  |  |  |  |
|  | -17,-59,64 | -0.29 | -2.06 | 0.050 | 0.502 |
| Betweenness Centrality | network | 0.01 | 0.92 | 0.366 |  |
| Average Path Length | network | 0.27 | 1.59 | 0.123 |  |
|  |  |  |  |  |  |
| Clustering Coefficient | network | -0.09 | -1.15 | 0.260 |  |
|  |  |  |  |  |  |
| Degree | network | -0.26 | -2.05 | 0.050 |  |
|  |  |  |  |  |  |

MNI = Montreal Neurological Institute; *β* = beta value; *t* = *t*-statistic; *d.f.* = degrees of freedom; p-unc. = p-uncorrected at <0.05; p-FDR = p-False Discovery Rate set at <0.05; and, “network” = statistical test comparing entire network across two groups.

**Stability of graph theoretic analyses**

The DN was assessed using network edges based on cost (one-sided positive and with an analysis threshold p-FDR <0.05 (two-sided). Results replicate those obtained when the DN was assessed using a network edges (adjacency matrix threshold) defined with *z*-score > 0.84, in terms of which nodes were nodes and the graph theoretic measures that were altered. The cost threshold can contain plausible information about functional brain networks (Dennis et al., 2012). The results of equivalent stability analyses of the somatomotor network, salience network, ventral attention network, dorsal attention networks, and the visual network are reported in **Supplementary files 1i-1m**.

**Supplementary file 1h. Results from graph theoretic stability analyses of default network topology.** The amnesic group and the control group were assessed using an adjacency matrix threshold based on cost. The analysis threshold (i.e., p-FDR <0.05, two-sided) was same as when the default network was assessed using the *z*-score based adjaency matrix threshold. Nodes that were not significant at the FDR-corrected analysis threshold applied to infer significance are reported by applying an uncorrected analysis threshold (p*-*uncorrected <0.05, two-sided).

| Analysis measure | ROI | *β* | *t* | p-unc | p-FDR |
| --- | --- | --- | --- | --- | --- |
|  |  |  |  |  |  |
|  |  |  |  |  |  |
| Global Efficiency | network | -0.00 | -2.10 | 0.045 |  |
|  |  |  |  |  |  |
|  | Left LTC | -0.13 | -2.21 | 0.036 | 0.541 |
|  |  |  |  |  |  |
| Local Efficiency | network | 0.14 | 1.28 | 0.211 |  |
|  |  |  |  |  |  |
|  | Left PCC | 0.41 | 2.57 | 0.021 | 0.098 |
|  |  |  |  |  |  |
|  | Left PHC | 0.73 | 8.62 | <0.000 | *0.001 |
|  |  |  |  |  |  |
|  | Left Rsp | 0.40 | 2.68 | 0.017 | 0.098 |
|  |  |  |  |  |  |
|  | Right Rsp | 0.54 | 3.41 | 0.004 | *0.034 |
|  |  |  |  |  |  |
| Betweenness Centrality | network | -0.04 | -2.63 | 0.014 |  |
|  |  |  |  |  |  |
|  | Left LTC | -0.06 | -2.42 | 0.023 | 0.262 |
|  |  |  |  |  |  |
|  | Left PCC | -0.13 | -2.35 | 0.026 | 0.262 |
|  |  |  |  |  |  |
| Average Path Length | network | -0.67 | 0.00 | 0.006 |  |
|  |  |  |  |  |  |
|  | Left PHC | -1.37 | -3.87 | 0.001 | *0.015 |
|  |  |  |  |  |  |
|  | Right PHC | -1.30 | -3.16 | 0.004 | *0.037 |
|  |  |  |  |  |  |
|  | Left HF | -0.94 | -2.45 | 0.022 | *0.049 |
|  |  |  |  |  |  |
|  | Right HF | -1.04 | -2.60 | 0.015 | *0.038 |
|  |  |  |  |  |  |
|  | Left Rsp | -0.62 | -2.69 | 0.012 | *0.037 |
|  |  |  |  |  |  |
|  | vmPFC | -0.77 | -2.70 | 0.012 | *0.040 |
|  |  |  |  |  |  |
|  | Left TempP | -1.07 | -2.70 | 0.012 | *0.037 |
| Clustering Coefficient | network | 0.08 | 1.29 | 0.209 |  |
|  |  |  |  |  |  |
| Degree | network | -0.00 | -3.74 | 0.001 |  |
|  |  |  |  |  |  |

ROI = region-of-interest/node; *β* = beta value; *t* = *t*-statistic; *d.f.* = degrees of freedom; p-unc. = p-uncorrected at <0.05; p-FDR = p-False Discovery Rate set at <0.05; and, “network” = statistical test comparing entire network across two groups. Left PCC = left posterior cingulate cortex; Right PCC = right posterior cingulate cortex; Left PHC = left parahippocampal cortex; Right PHC = right parahippocampal cortex; Left Rsp = left retrosplenial; Right Rsp = right retrosplenial cortex; Left HF = left hippocampal formation; Right HF = right hippocampal formation; vmPFC = ventromedial prefrontal cortex; and Left TempP = left temporal pole. Network edges (adjacency matrix threshold): cost = 0.15 (one-sided, positive).

**Supplementary file 1i. Results from graph theoretic stability analyses of somatomotor network topology.** The amnesic group and the control group were assessed using an adjacency matrix threshold based on cost. The analysis threshold (i.e., p-FDR <0.05, two-sided) was same as when the somatomotor network was assessed using the *z*-score based adjaency matrix threshold. Nodes that were not significant at the p*-*FDR-corrected analysis threshold applied to infer significance are reported by applying an uncorrected analysis threshold (p*-*uncorrected <0.05, two-sided).

| Analysis measure | MNI  co-ordinates (x,y,z) | *β* | *t* | p-unc. | p-FDR |
| --- | --- | --- | --- | --- | --- |
|  |  |  |  |  |  |
|  |  |  |  |  |  |
| Global Efficiency | network | -0.03 | -1.38 | 0.180 |  |
|  |  |  |  |  |  |
|  | -7, -21, 65 | -0.06 | -2.77 | 0.010 | 0.124 |
|  |  |  |  |  |  |
|  | -13, -17, 75 | -0.16 | -2.58 | 0.016 | 0.124 |
|  |  |  |  |  |  |
|  | -21, -31, 61 | -0.07 | -2.61 | 0.015 | 0.124 |
|  |  |  |  |  |  |
|  | -23, -30, 72 | -0.08 | -2.54 | 0.017 | 0.124 |
|  |  |  |  |  |  |
| Local Efficiency | network | 0.03 | 0.79 | 0.434 |  |
|  | 0, -15, 47 | 0.25 | 3.17 | 0.004 | 0.124 |
| Betweenness Centrality | network | -0.01 | -0.99 | 0.333 |  |
| Average Path Length | network | -0.17 | -0.83 | 0.412 |  |
|  |  |  |  |  |  |
| Clustering Coefficient | network | 0.02 | 0.81 | 0.426 |  |
|  |  |  |  |  |  |
|  | -0, -15, 47 | 0.24 | 3.00 | 0.006 | 0.178 |
|  |  |  |  |  |  |
| Degree | network | -0 | -5.19 | <0.0001 |  |
|  |  |  |  |  |  |
|  | -13, -17, 75 | -1.96 | -2.07 | 0.048 | 0.327 |
|  |  |  |  |  |  |
|  | -23, -30, 72 | -2.09 | -2.39 | 0.024 | 0.327 |
|  |  |  |  |  |  |
|  | 2, -28, 60 | 2.05 | 2.16 | 0.040 | 0.327 |
|  |  |  |  |  |  |
|  | 47, -30, 49 | 1.88 | 2.30 | 0.030 | 0.327 |
|  |  |  |  |  |  |

MNI = Montreal Neurological Institute; *β* = beta value; *t* = *t*-statistic; *d.f.* = degrees of freedom; p-unc. = p-uncorrected at <0.05; p-FDR = p-False Discovery Rate set at <0.05 and, “network” = statistical test comparing entire network across two groups. Network edges (adjacency matrix threshold): cost = 0.15 (one-sided, positive).

**Supplementary file 1j. Results from graph theoretic stability analyses of visual network topology.** The amnesic group and the control group were assessed using an adjacency matrix threshold based on cost. The analysis threshold (i.e., p-FDR <0.05, two-sided) was same as when the visual network was assessed using the *z*-score based adjaency matrix threshold. Nodes that were not significant at the p*-*FDR-corrected analysis threshold applied to infer significance are reported by applying an uncorrected analysis threshold (p*-*uncorrected <0.05, two-sided).

| Analysis measure | MNI  co-ordinates  (x,y,z) | *β* | *t* | p-unc. | p-FDR |
| --- | --- | --- | --- | --- | --- |
|  |  |  |  |  |  |
|  |  |  |  |  |  |
| Global Efficiency | network | 0.01 | 0.41 | 0.685 |  |
|  |  |  |  |  |  |
| Local Efficiency | network | 0.03 | 0.86 | 0.395 |  |
|  |  |  |  |  |  |
|  | -47, -76, 10 | 0.28 | 2.54 | 0.017 | 0.332 |
|  |  |  |  |  |  |
|  | -18, -68, 5 | 0.27 | 2.45 | 0.023 | 0.332 |
|  |  |  |  |  |  |
| Betweenness Centrality | network | 0.01 | 0.79 | 0.437 |  |
| Average Path Length | network | 0.19 | 1.09 | 0.287 |  |
|  |  |  |  |  |  |
| Clustering Coefficient | network | 0.02 | 0.62 | 0.542 |  |
|  |  |  |  |  |  |
|  | -18, -68, 5 | 0.25 | 2.58 | 0.017 | 0.347 |
|  |  |  |  |  |  |
|  | -47, -76, -10 | 0.24 | 2.40 | 0.024 | 0.347 |
|  |  |  |  |  |  |
| Degree | network | -0.00 | -5.19 | <0.0001 |  |
|  |  |  |  |  |  |

MNI = Montreal Neurological Institute; *β* = beta value; *t* = *t*-statistic; *d.f.* = degrees of freedom; p-unc. = p-uncorrected at <0.05; p-FDR = p-False Discovery Rate set at <0.05 and, “network” = statistical test comparing entire network across two groups. Network edges (adjacency matrix threshold): cost = 0.15 (one-sided, positive).

**Supplementary file 1k. Results from graph theoretic stability analyses of the salience network**. Amnesic group and the control group were assessed using an adjacency matrix threshold based on cost. The analysis threshold (i.e., p-FDR <0.05, two-sided) was same as when the salience network was assessed using the *z*-score based adjaency matrix threshold. Nodes that were not significant at the p*-*FDR-corrected analysis threshold applied to infer significance are reported by applying an uncorrected analysis threshold (p*-*uncorrected <0.05, two-sided).

| Analysis measure | MNI  co-ordinates  (x,y,z) | *β* | *t* | p-unc. | p-FDR |
| --- | --- | --- | --- | --- | --- |
|  |  |  |  |  |  |
|  |  |  |  |  |  |
| Global Efficiency | network | 0.02 | 0.83 | 0.413 |  |
|  |  |  |  |  |  |
| Local Efficiency | network | -0.02 | -0.52 | 0.607 |  |
| Betweenness Centrality | network | 0.01 | 0.57 | 0.591 |  |
|  |  |  |  |  |  |
|  | 34, 16, -8 | 0.06 | 2.22 | 0.035 | 0.550 |
| Average Path Length | network | 0.12 | 0.49 | 0.625 |  |
|  |  |  |  |  |  |
| Clustering Coefficient | network | -0.02 | -0.46 | 0.646 |  |
|  |  |  |  |  |  |
| Degree | network | 0.00 | 3.74 | 0.001 |  |
|  |  |  |  |  |  |
|  | 37, 32, -2 | -0.96 | -2.41 | 0.023 | 0.414 |
|  |  |  |  |  |  |

MNI = Montreal Neurological Institute; *β* = beta value; *t* = *t*-statistic; *d.f.* = degrees of freedom; p-unc. = p-uncorrected at <0.05; p-FDR = p-False Discovery Rate set at <0.05 and, “network” = statistical test comparing entire network across two groups. Network edges (adjacency matrix threshold): cost = 15 (one-sided, positive).

**Supplementary file 1l. Results from graph theoretic stability analyses of the ventral attention network**. The amnesic group and the control group were assessed using an adjacency matrix threshold based on cost. The analysis threshold (i.e., p-FDR <0.05, two-sided) was same as when the ventral attention network was assessed using the *z*-score based adjaency matrix threshold. Nodes that were not significant at the p*-*FDR-corrected analysis threshold applied to infer significance are reported by applying an uncorrected analysis threshold (p*-*uncorrected <0.05, two-sided).

| Analysis measure | MNI  co-ordinates  (x,y,z) | *β* | *t* | p-unc. | p-FDR |
| --- | --- | --- | --- | --- | --- |
|  |  |  |  |  |  |
| Global Efficiency | network | -0.02 | -1.25 | 0.224 |  |
|  |  |  |  |  |  |
|  | -49, 25 ,-1 | -0.12 | -2.44 | 0.022 | 0.215 |
|  |  |  |  |  |  |
|  | -10, 11, 67 | -0.08 | -2.20 | 0.045 | 0.226 |
|  |  |  |  |  |  |
| Local Efficiency | network | 0.23 | 2.18 | 0.039 |  |
|  |  |  |  |  |  |
|  | -56, -50, 10 | 0.41 | 2.49 | 0.021 | 0.084 |
|  |  |  |  |  |  |
|  | 56, -46, 11 | 0.39 | 2.64 | 0.015 | 0.084 |
| Betweenness Centrality | network | -0.02 | -1.57 | 0.127 |  |
| Average Path Length | network | -0.19 | -1.32 | 0.199 |  |
|  |  |  |  |  |  |
|  | -55, -40, 14 | -0.47 | -2.09 | 0.049 | 0.349 |
|  |  |  |  |  |  |
| Clustering Coefficient | network | 0.21 | 2.09 | 0.047 |  |
|  |  |  |  |  |  |
|  | -56, -50, 10 | 0.41 | 2.49 | 0.021 | 0.084 |
|  |  |  |  |  |  |
|  | 56, -46, 11 | 0.37 | 2.52 | 0.019 | 0.084 |
|  |  |  |  |  |  |
| Degree | network | -0.00 | -4.88 | <0.000 |  |
|  |  |  |  |  |  |
|  | -49, 25, -1 | -0.63 | -2.13 | 0.042 | 0.421 |
|  |  |  |  |  |  |

MNI = Montreal Neurological Institute; *β* = beta value; *t* = *t*-statistic; *d.f.* = degrees of freedom; p-unc. = p-uncorrected at <0.05; p-FDR = p-False Discovery Rate set at <0.05 and, “network” = statistical test comparing entire network across two groups. Network edges (adjacency matrix threshold): cost = 0.15 (one-sided, positive).

**Supplementary file 1m. Results from graph theoretic stability analyses of the dorsal attention network**. Amnesic group and the control group were assessed using an adjacency matrix threshold based on cost. The analysis threshold (i.e., p-FDR <0.05, two-sided) was same as when the dorsal attention network was assessed using the *z*-score based adjaency matrix threshold. Nodes that were not significant at the p*-*FDR-corrected analysis threshold applied to infer significance are reported by applying an uncorrected analysis threshold (p*-*uncorrected <0.05, two-sided).

| Analysis measure | MNI  co-ordinate  (x,y,z) | *β* | *t* | p-unc. | p-FDR |
| --- | --- | --- | --- | --- | --- |
|  |  |  |  |  |  |
|  |  |  |  |  |  |
| Global Efficiency | network | -0.02 | 1.41 | 0.169 |  |
|  |  |  |  |  |  |
| Local Efficiency | network | -0.01 | -0.11 | 0.914 |  |
| Betweenness Centrality | network | 0.02 | 1.48 | 0.150 |  |
| Average Path Length | network | 0.23 | 1.45 | 0.160 |  |
|  |  |  |  |  |  |
|  | 10, -62, 61 | 0.45 | 2.09 | 0.047 | 0.257 |
|  |  |  |  |  |  |
|  | 25, -58, 60 | 0.38 | 2.09 | 0.046 | 0.257 |
|  |  |  |  |  |  |
| Clustering Coefficient | network | -0.01 | -0.10 | 0.923 |  |
|  |  |  |  |  |  |
| Degree | network | -0.00 | -3.74 | <0.001 |  |
|  |  |  |  |  |  |

MNI = Montreal Neurological Institute; *β* = beta value; *t* = *t*-statistic; *d.f.* = degrees of freedom; p-unc. = p-uncorrected at <0.05; p-FDR = p-False Discovery Rate set at <0.05 and, “network” = statistical test comparing entire network across two groups. Network edges (adjacency matrix threshold): cost = 0.15 (one-sided, positive).

**Co-ordinates for control networks graph theoretic analyses**

**Supplementary file 1n. MNI co-ordinates for the nodes used in control network analyses.** MNI co-ordinates for the nodes that correspond to the somatomotor network, visual network, salience network, dorsal attention network, and ventral attention network were based on the parcellation scheme proposed by Power et al. (2011)**.** Probabilistic anatomical locations of the MNI co-ordinates are defined using the Harvard-Oxford Cortical and Subcortical Probabilistic Atlases.

| Resting-state network | |  | | MNI  co-ordinates | | |  | | Regions | | |
| --- | --- | --- | --- | --- | --- | --- | --- | --- | --- | --- | --- |
|  | | x | | y | | | z |  | | | |
| Sensory/  somatomotor hand | | -7 | | - 52 | | | 61 | Precuneus (60%) | | | |
|  | | -14 | | -18 | | | 40 | Precentral gyrus (24%) | | | |
|  | | 0 | | -15 | | | 47 | Precentral gyrus (23%) | | | |
|  | | 10 | | -2 | | | 45 | Juxtapositional lobule cortex 52%) | | | |
|  | | -7 | | -21 | | | 65 | Precentral gyrus (26%) | | | |
|  | | -7 | | -33 | | | 72 | Precentral gyrus (24%), postcentral gyrus (20%) | | | |
|  | | 13 | | -33 | | | 75 | Postcentral gyrus (58%) | | | |
|  | | -54 | | -23 | | | 43 | Postcentral gyrus (58%) | | | |
|  | | 29 | | -17 | | | 71 | Precentral gyrus (54%) | | | |
|  | | 10 | | -46 | | | 73 | Postcentral gyrus (36%) | | | |
|  | | -23 | | -30 | | | 72 | Postcentral gyrus (38%) | | | |
|  | | -40 | | -19 | | | 54 | Precentral gyrus (46%) | | | |
|  | | 29 | | -39 | | | 59 | Postcentral gyrus (41%) | | | |
|  | | 50 | | -20 | | | 42 | Postcentral gyrus (53%) | | | |
|  | | -38 | | -27 | | | 69 | Postcentral gyrus (51%) | | | |
|  | |  | |  | | |  |  | | | |
|  | | 20 | | -29 | | | 60 | Precentral gyrus | | | |
|  | |  | |  | | |  |  | | | |
|  | | 44 | | -8 | | | 57 | Precentral gyrus (57%) | | | |
|  | |  | |  | | |  |  | | | |
|  | | -29 | | -43 | | | 61 | Superior parietal lobule | | | |
|  | |  | |  | | |  |  | | | |
|  | | 10 | | -17 | | | 74 | Precentral gyrus | | | |
|  | |  | |  | | |  |  | | | |
|  | | 22 | | -42 | | | 69 | Superior parietal lobule (34%), postcentral gyrus (32%) | | | |
|  | |  | |  | | |  |  | | | |
|  | | -45 | | -32 | | | 47 | Postcentral gyrus (42%) | | | |
|  | |  | |  | | |  |  | | | |
|  | | -21 | | -31 | | | 61 | Postcentral gyrus (39%) | | | |
|  | |  | |  | | |  |  | | | |
|  | | -13 | | -17 | | | 75 | Precentral gyrus (44%) | | | |
|  | |  | |  | | |  |  | | | |
|  | | 42 | | -20 | | | 55 | Precentral gyrus (39%) | | | |
|  | |  | |  | | |  |  | | | |
|  | | -38 | | -15 | | | 69 | Precentral gyrus (45%) | | | |
|  | |  | |  | | |  |  | | | |
|  | | -16 | | -46 | | | 73 | Postcentral gyrus (42%) | | | |
|  | |  | |  | | |  |  | | | |
|  | | 2 | | -28 | | | 60 | Precentral gyrus (51%) | | | |
|  | |  | |  | | |  |  | | | |
|  | | 3 | | -17 | | | 58 | Precentral gyrus (65%) | | | |
|  | |  | |  | | |  |  | | | |
|  | | 38 | | -17 | | | 45 | Precentral gyrus (50%) | | | |
|  | |  | |  | | |  |  | | | |
|  | | -49 | | -11 | | | 35 | Precentral gyrus (39%) | | | |
|  | |  | |  | | |  |  | | | |
|  | | 36 | | -9 | | | 14 | Insular cortex (53%) | | | |
|  | |  | |  | | |  |  | | | |
|  | | 51 | | -6 | | | 32 | Precentral gyrus (36%) | | | |
|  | |  | |  | | |  |  | | | |
|  | | -53 | | -10 | | | 24 | Postcentral gyrus (30%) | | | |
|  | |  | |  | | |  |  | | | |
|  | | 66 | | -8 | | | 25 | Postcentral gyrus (70%) | | | |
|  | |  | |  | | |  |  | | | |
|  | | 47 | | -30 | | | 49 | Postcentral gyrus (47%) | | | |
|  | |  | |  | | |  |  | | | |
|  | |  | |  | | |  |  | | | |
| Visual | |  | |  | | |  |  | | | |
|  | | 18 | | -47 | | | -10 | Lingual gyrus (89%) | | | |
|  | |  | |  | | |  |  | | | |
|  | | 40 | | -72 | | | 14 | Lateral occipital cortex (29%) | | | |
|  | |  | |  | |  | | | | |  |
|  | | 8 | | -72 | | | 11 | Intracalcarine cortex (64%) | | | |
|  | |  | |  | | |  |  | | | |
|  | | -8 | | -81 | | | 7 | Intracalcarine cortex (65%) | | | |
|  | |  | |  | | |  |  | | | |
|  | | -28 | | -79 | | | 19 | Lateral occipital cortex (59%) | | | |
|  | |  | |  | | |  |  | | | |
|  | | 20 | | -66 | | | 2 | Intracalcarine cortex (24%) | | | |
|  | |  | |  | | |  |  | | | |
|  | | -24 | | -91 | | | 19 | Occipital pole (39%) | | | |
|  | |  | |  | | |  |  | | | |
|  | | 27 | | -59 | | | -9 | Temporal occipital fusiform cortex (29%) | | | |
|  | | -15 | | -72 | | | -8 | Lingual gyrus (44%) | | | |
|  | |  | |  | | |  |  | | | |
|  | | -18 | | -68 | | | 5 | Intracalcarine cortex (53%) | | | |
|  | |  | |  | | |  |  | | | |
|  | | 43 | | -78 | | | -12 | Lateral occipital cortex (60%) | | | |
|  | |  | |  | | |  |  | | | |
|  | | -47 | | -76 | | | -10 | Lateral occipital cortex, inferior division (84%) | | | |
|  | |  | |  | | |  |  | | | |
|  | | -14 | | -91 | | | 31 | Occipital lobe (51%) | | | |
|  | |  | |  | | |  |  | | | |
|  | | 15 | | -87 | | | 37 | Occipital pole (33%) | | | |
|  | |  | |  | | |  |  | | | |
|  | | 29 | | -77 | | | 25 | Lateral occipital cortex, superior division (68%) | | | |
|  | |  | |  | | |  |  | | | |
|  | | 20 | | -86 | | | -2 | Lingual gyrus (13%), occipital pole (13%), occipital fusiform gyrus (13%) | | | |
|  | |  | |  | | |  |  | | | |
|  | | 15 | | -77 | | | 31 | Cuneal cortex (49%) | | | |
|  | |  | |  | | |  |  | | | |
|  | | -16 | | -52 | | | -1 | Lingual gyrus (56%) | | | |
|  | |  | |  | | |  |  | | | |
|  | | 42 | | -66 | | | -8 | Lateral occipital cortex, inferior division (39%) | | | |
|  | |  | |  | | |  |  | | | |
|  | | 24 | | -87 | | | 24 | Lateral occipital cortex (40%) | | | |
|  | |  | |  | | |  |  | | | |
|  | | 6 | | -72 | | | 24 | Cuneal cortex (45%) | | | |
|  | |  | |  | | |  |  | | | |
|  | | -42 | | -74 | | | 0 | Lateral occipital cortex, inferior division (64%) | | | |
|  | |  | |  | | |  |  | | | |
|  | | 26 | | -79 | | | -16 | Occipital fusiform gyrus (69%) | | | |
|  | |  | |  | | |  |  | | | |
|  | | -16 | | -77 | | | 34 | Cuneal cortex (32%) | | | |
|  | |  | |  | | |  |  | | | |
|  | | -3 | | -81 | | | 21 | Cuneal cortex (47%) | | | |
|  | |  | |  | | |  |  | | | |
|  | | -40 | | -88 | | | -6 | Lateral occipital cortex, inferior division (64%) | | | |
|  | |  | |  | | |  |  | | | |
|  | | 37 | | -84 | | | 13 | Superior occipital cortex, superior division (38%) | | | |
|  | |  | |  | | |  |  | | | |
|  | | 6 | | -81 | | | 6 | Intracalcarine cortex (51%) | | | |
|  | |  | |  | | |  |  | | | |
|  | | -26 | | -90 | | | 3 | Lateral occipital cortex, inferior division (20%) | | | |
|  | |  | |  | | |  |  | | | |
|  | | -33 | | -79 | | | -13 | Occipital fusiform gyrus (46%) | | | |
|  | |  | |  | | |  |  | | | |
|  | | 37 | | -81 | | | 1 | Lateral occipital cortex, inferior division (45%) | | | |
|  | |  | |  | | |  |  | | | |
|  | | -44 | | 2 | | | 46 | Precentral gyrus (44%) | | | |
|  | |  | |  | | |  |  | | | |
| Salience | |  | |  | | |  |  | | | |
|  | | 11 | | -39 | | | 50 | Postcentral gyrus (27%), precuneus cortex (26%) | | | |
|  | |  | |  | | |  |  | | | |
|  | | 55 | | -45 | | | 37 | Supramarginal gyrus, posterior division (44%) | | | |
|  | |  | |  | | |  |  | | | |
|  | | 42 | | 0 | | | 47 | Precentral gyrus (33%) | | | |
|  | |  | |  | | |  |  | | | |
|  | | 31 | | 33 | | | 26 | Middle frontal gyrus (31%) | | | |
|  | |  | |  | | |  |  | | | |
|  | | 48 | | 22 | | | 10 | Inferior frontal gyrus pars triangularis (28%), inferior frontal gyrus pars opercularis (25%) | | | |
|  | |  | |  | | |  |  | | | |
|  | | -35 | | 20 | | | 0 | Insular cortex (76%) | | | |
|  | |  | |  | | |  |  | | | |
|  | | 36 | | 22 | | | 3 | Insular cortex (47%) | | | |
|  | |  | |  | | |  |  | | | |
|  | | 37 | | 32 | | | -2 | Inferior frontal gyrus, pars triangularis (15%), frontal orbital cortex (13%) | | | |
|  | |  | |  | | |  |  | | | |
|  | | 34 | | 16 | | | -8 | Insular cortex (86%) | | | |
|  | |  | |  | | |  |  | | | |
|  | | -11 | | 26 | | | 25 | Cingulate gyrus, anterior division (31%) | | | |
|  | |  | |  | | |  |  | | | |
|  | | -1 | | 15 | | | 44 | Paracingulate gyrus (63%) | | | |
|  | |  | |  | | |  |  | | | |
|  | | -28 | | 52 | | | 21 | Frontal pole | | | |
|  | |  | |  | | |  |  | | | |
|  | | 0 | | 30 | | | 27 | Cingulate gyrus, anterior division (50%) | | | |
|  | |  | |  | | |  |  | | | |
|  | | 5 | | 23 | | | 37 | Paracingulate gyrus (54%) | | | |
|  | |  | |  | | |  |  | | | |
|  | | 10 | | 22 | | | 27 | Cingulate gyrus, anterior division (44%) | | | |
|  | |  | |  | | |  |  | | | |
|  | | 31 | | 56 | | | 14 | Frontal pole (88%) | | | |
|  | |  | |  | | |  |  | | | |
|  | | 26 | | 50 | | | 27 | Frontal pole (71%) | | | |
|  | |  | |  | | |  |  | | | |
|  | | -39 | | 51 | | | 17 | Frontal pole (82%) | | | |
|  | |  | |  | | |  |  | | | |
|  | |  | |  | | |  |  | | | |
| Ventral Attention | |  | |  | | |  |  | | | |
|  | | -10 | | 11 | | | 67 | Superior frontal gyrus (56%) | | | |
|  | |  | |  | | |  |  | | | |
|  | | 54 | | -43 | | | 22 | Supramarginal gyrus, posterior division (33%) | | | |
|  | |  | |  | | |  |  | | | |
|  | | -56 | | -50 | | | 10 | Supramarginal gyrus, posterior division (26%), middle temporal gyrus, temporooccipital part (24%) | | | |
|  | |  | |  | | |  |  | | | |
|  | | -55 | | -40 | | | 14 | Planum temporale (27%) | | | |
|  | |  | |  | | |  |  | | | |
|  | | 52 | | -33 | | | 8 | Superior temporal gyrus, posterior division (14%) | | | |
|  | |  | |  | | |  |  | | | |
|  | | 51 | | -29 | | | -4 | Middle temporal gyrus, posterior division (55%) | | | |
|  | |  | |  | | |  |  | | | |
|  | | 56 | | -46 | | | 11 | Middle temporal gyrus, temporooccipital part (34%) | | | |
|  | |  | |  | | |  |  | | | |
|  | | 53 | | 33 | | | 1 | Inferior frontal gyrus, pars triangularis (46%) | | | |
|  | |  | |  | | |  |  | | | |
|  | | -49 | | 25 | | | -1 | Inferior frontal gyrus, pars triangularis (43%) | | | |
|  | |  | |  | | |  |  | | | |
| Dorsal Attention | |  | |  | | |  |  | | | |
|  | | 10 | | -62 | | | 61 | Lateral occipital cortex, superior division (37%) | | | |
|  | |  | |  | | |  |  | | | |
|  | | -52 | | -63 | | | 5 | Lateral occipital cortex, inferior division (34%) | | | |
|  | |  | |  | | |  |  | | | |
|  | | 22 | | -65 | | | 48 | Lateral occipital cortex, superior division (35%) | | | |
|  | |  | |  | | |  |  | | | |
|  | | 46 | | -59 | | | 4 | Middle temporal gyrus, temporooccipital part (36%) | | | |
|  | |  | |  | | |  |  | | | |
|  | | 25 | | -58 | | | 60 | Lateral occipital cortex, superior division (33%) | | | |
|  | |  | |  | | |  |  | | | |
|  | | -33 | | -46 | | | 47 | Superior parietal lobule (35%) | | | |
|  | |  | |  | | |  |  | | | |
|  | | -27 | | -71 | | | 37 | Lateral occipital cortex, superior division (76%) | | | |
|  | |  | |  | | |  |  | | | |
|  | | -32 | | -1 | | | 54 | Middle frontal gyrus (41%) | | | |
|  | |  | |  | | |  |  | | | |
|  | | -42 | | -60 | | | -9 | Inferior temporal gyrus, temporooccipital part (31%) | | | |
|  | |  | |  | | |  |  | | | |
|  | | -17 | | -59 | | | 64 | Lateral occipital cortex, superior division (38%) | | | |
|  | |  | |  | | |  |  | | | |
|  | | 29 | | -5 | | | 54 | Precentral gyrus (20%), middle frontal gyrus (20%), superior frontal gyrus (20%) | | | |
|  | |  | |  | | |  |  | | | |
|  | |  | |  | | |  |  | | | |
|  |  | |  | |  | | | | |  |  |

**Seed-to-voxel analyses**

**Supplementary file 1o. Results from between-group seed-to-voxel functional connectivity based analyses.** No brain regions exhibited significant differences (height threshold, p-uncorrected <0.001 and an extent threshold of p-FDR<0.05 at the cluster level) in functional connectivity between the amnesic group and the control group, when tested with left and right hippocampal seed regions-of-interest, an occipital pole seed within the visual network, and a seed in primary motor cortex within the somatomotor network. Seed regions were spheres with 8 mm radii. For the left hippocampal seed region, a between-group difference in functional connectivity was found only at a lenient, *p*-uncorrected <0.05 cluster-size threshold. There were no significant clusters for the right hippocampal seed region even when between-group differences were assessed at a cluster-size threshold set at *p*-uncorrected <0.05 (see **Figure 7 – figure supplement 1**).

| Seed region-of-interest  (MNI co-ordinates) | Clusters | | | | |
| --- | --- | --- | --- | --- | --- |
|  | MNI  co-ordinates for peak voxel of cluster  (x, y, z) | size | size p-unc | size p-FDR | |
|  |  |  |  |  | |
| Left hippocampus | -2, 2, 8 | 44 | 0.015 | 0.322 | |
| (-24, -22, 16) |  |  |  | |  |
|  |  |  |  | |  |
|  |  |  |  |  | |
|  |  |  |  |  | |
| Right hippocampus | - | - | - | - | |
| (24, -22, -16) |  |  |  | |  |
|  |  |  |  | |  |
|  |  |  |  |  | |
| Occipital pole | 54, 0, -24 | 41 | 0.034 | 0.171 | |
| (18, -47, -10) |  |  |  | |  |
|  |  |  |  | |  |
|  |  |  |  |  | |
| Motor Cortex (M1) | - | - | - | - | |
| (-40, -19, 54) |  |  |  |  | |
|  |  |  |  |  | |
|  |  |  |  |  | |
|  |  |  |  |  | |

MNI = Montreal Neurological Institute; size = cluster size; *t* = *t*-statistic; *d.f.* = degrees of freedom; size p-unc. = cluster-size threshold p-uncorrected at <0.05; cluster-size p-FDR = p-False Discovery Rate set at <0.05; “ - “ = no clusters identified. Height threshold, p-uncorrected <0.001 (two-sided).

**Supplementary file 1p. Results from left and right hippocampus seed-to-voxel functional connectivity based analyses.** MNI co-ordinates of brain regions that exhibited significant functional connectivity with left and right hippocampal seed regions-of-interest shown separately for the amnesic group and the control group (height threshold, p-uncorrected <0.001 and an extent threshold of p-FDR <0.05 at the cluster level). Seed regions were spheres with 8 mm radii (see **Figure 7 – figure supplement 2**).

| Seed region-of-interest  (MNI co-ordinates) | | Clusters | | | | | | | |
| --- | --- | --- | --- | --- | --- | --- | --- | --- | --- |
|  | | MNI  co-ordinates for peak voxel of cluster  (x, y, z) | | size | | size p-unc | | size p-FDR | |
|  | |  | |  | |  | |  | |
|  |  | |  | |  | |  |  |  |
|  |  | |  | |  | |  |  |  |
| *Amnesic group* |  | |  | |  | |  |  |  |
| Left hippocampus | | -22, -18, -18 | | 7087 | | <0.0001 | | <0.0001 | |
| (-24, -22, 16) | | -10, -88, -18 | | 224 | | <0.0001 | | <0.0001 | |
|  | |  | |  | |  | |  | |
| Right hippocampus | | 22, -24, -16 | | 7826 | | <0.0001 | | <0.0001 | |
| (24, -22, -16) | | -24, -18, -14 | | 1881 | | <0.0001 | | <0.0001 | |
|  | | 6, 48, 42 | | 187 | | <0.0001 | | 0.0002 | |
|  | | 22, 66, 22 | | 125 | | <0.0001 | | 0.0030 | |
|  | | -2, -42, 22 | | 103 | | 0.0002 | | 0.0096 | |
|  | | 2, -14, -42 | | 101 | | 0.0002 | | 0.0107 | |
|  | | -18, 42, 2 | | 65 | | 0.0019 | | 0.0866 | |
|  | |  | |  | |  | |  | |
| *Control group* | |  | |  | |  | |  | |
|  | |  | |  | |  | |  | |
| Left hippocampus | | 22, -20, -18 | | 11714 | | <0.0001 | | <0.0001 | |
| (-24, -22, 16) | | 0, -56, -36 | | 244 | | <0.0001 | | <0.0001 | |
|  | | 6, -44, 68 | | 79 | | 0.0004 | | 0.0153 | |
|  | |  | |  | |  | |  | |
|  | |  | |  | |  | | |  |
| Right hippocampus | | 26, -20, 16 | | 8020 | | <0.0001 | | <0.0001 | |
| (24, -22, -16) | | -24, -18, -14 | | 3131 | | <0.0001 | | <0.0001 | |
|  | | 48, -62, 50 | | 192 | | <0.0001 | | <0.0001 | |
|  | | -26, -12, 32 | | 96 | | 0.0001 | | 0.0034 | |
|  | | 18, -92, -12 | | 93 | | 0.0001 | | 0.0034 | |
|  | | -8, -56, -38 | | 78 | | 0.0004 | | 0.0076 | |
|  | | -18, -78, 0 | | 48 | | 0.0032 | | 0.0456 | |
|  | | -44, 56, -38 | | 48 | | 0.0032 | | 0.0456 | |
|  | | -62, 2, -26 | | 48 | | 0.0032 | | 0.0456 | |
|  | |  | |  | |  | |  | |

MNI = Montreal Neurological Institute; size = cluster size; size p-unc. = cluster-size threshold p-uncorrected at <0.05; cluster-size p-FDR = p-False Discovery Rate set at <0.05; Height threshold, p-uncorrected <0.001 (two-sided).

**Multiple regression analyses**

**Supplementary file 1q. Results from robust multiple linear regression analysis on nodes in the default network with significantly increased average path length (independent variables) relative to the control group and total internal (episodic) detail remembered on the AI.** Average path length values entered into the robust multiple regression analysis were based on the difference between each participant and the mean of the control group for each affected ROI/node.

| Independent variables | Regression coefficient | | | |
| --- | --- | --- | --- | --- |
|  | *β* | sem | *t* | p |
| Left PHC | 1.05 | 0.22 | 4.69 | *0.018 |
|  |  |  |  |  |
| Right PHC | 0.24 | 0.25 | 0.97 | 0.404 |
|  |  |  |  |  |
| Left HF | 1.81 | 0.23 | 7.87 | *0.004 |
|  |  |  |  |  |
| Right HF | -1.55 | 0.18 | -8.66 | *0.003 |
|  |  |  |  |  |
| Left Rsp | 26.61 | 2.12 | 12.55 | *0.001 |
|  |  |  |  |  |
| vmPFC | -26.31 | 2.28 | -11.55 | *0.001 |
|  |  |  |  |  |
| Left TempP | -1.70 | 0.166 | -10.26 | *0.002 |
|  |  |  |  |  |

Left PHC = left parahippocampal cortex; Right PHC = right parahippocampal cortex; Left HF = left hippocampal formation; Right HF = right hippocampal formation; Left Rsp = left retrosplenial cortex; Left TempP = left temporal pole; and vmPFC = ventromedial prefrontal cortex.

**Supplementary file 1r. Results from multiple linear regression analysis examining link between nodes in the DN that exhibited signficantly increased local efficiency and internal (episodic) detail remembered on the AI.** Local efficiency values entered into the robust multiple regression analysis were based on the difference between each participant and the mean of the control group for each affected ROI/node.

| Independent variables | Regression coefficient | | | |
| --- | --- | --- | --- | --- |
|  | *β* | sem | *t* | p |
|  |  |  |  |  |
| Left PHC | 0.41 | 0.61 | 0.66 | 0.529 |
|  |  |  |  |  |
| Right Rsp | -0.12 | 1.11 | -0.11 | 0.914 |
|  |  |  |  |  |
| Left PCC | 0.53 | 0.85 | 0.62 | 0.557 |
|  |  |  |  |  |

Left PHC = left parahippocampal cortex; Right Rsp = right retrosplenial cortex; Left PCC = left posterior cingulate cortex.
