# Supplementary material for: Human hippocampal CA3 damage disrupts both recent and remote episodic memories
Source: eLife. 2020 Jan 24;9:e41836. doi: 10.7554/eLife.41836 (PMC6980860; doi:10.7554/eLife.41836)
Supplement: Transparent reporting form [file elife-41836-transrepform.pdf]

eLIFE
1st Floor
24 Hills Road
Cambridge CB2 1JP, UK
P 01223 855340
W elifesciences.org
T @elife

eLife's transparent reporting form

We encourage authors to provide detailed information *within their submission* to facilitate the interpretation and replication of experiments. Authors can upload supporting documentation to indicate the use of appropriate reporting guidelines for health-related research (see EQUATOR Network), life science research (see the BioSharing Information Resource), or the ARRIVE guidelines for reporting work involving animal research. Where applicable, authors should refer to any relevant reporting standards documents in this form.

If you have any questions, please consult our Journal Policies and/or contact us: editorial@elifesciences.org.

Sample-size estimation

- You should state whether an appropriate sample size was computed when the study was being designed
- You should state the statistical method of sample size computation and any required assumptions
- If no explicit power analysis was used, you should describe how you decided what sample (replicate) size (number) to use

Please outline where this information can be found within the submission (e.g., sections or figure legends), or explain why this information doesn't apply to your submission:

Sample size was based on standards in the literature for studies involving participants with rare focal lesions involving the hippocampus and medial temporal lobe (Materials and Methods, Participants).

Replicates

- You should report how often each experiment was performed
- You should include a definition of biological versus technical replication
- The data obtained should be provided and sufficient information should be provided to indicate the number of independent biological and/or technical replicates
- If you encountered any outliers, you should describe how these were handled
- Criteria for exclusion/inclusion of data should be clearly stated
- High-throughput sequence data should be uploaded before submission, with a private link for reviewers provided (these are available from both GEO and ArrayExpress)

Please outline where this information can be found within the submission (e.g., sections or figure legends), or explain why this information doesn't apply to your submission:

eLife Sciences Publications, Ltd is a limited liability non-profit non-stock corporation incorporated in the State of Delaware, USA, with company number 5030732, and is registered in the UK with company number FC030576 and branch number BR015634 at the address 1st Floor, 24 Hills Road, Cambridge CB2 1JP | August 2014

Two independent raters scored 100% of the episodes acquired from each time period for all participants in the amnesia and control groups (Results; Material and Methods).
The results reported in the subsections on quantitative hippocampal morphometry, involving subset of participants, replicate those reported in our previous study which involved 18 participants (Miller et al., 2017).
Graph theoretic analyses were replicated using different thresholding criteria to establish reliability of the effects on the DN (Results, Stability of Effects).
Participant inclusion/exclusion criteria are provided in Materials and Methods section and Participants section.
Criteria for the inclusion of data are reported under the relevant Materials and Methods subsections and Results section (Materials and Methods, Neuropsychological assessment; Materials and Methods, 7.0-Tesla magnetic resonance image acquisition and protocols)
Multiple regression was conducted using Huber's method of correction for outliers.

Statistical reporting

- Statistical analysis methods should be described and justified
- Raw data should be presented in figures whenever informative to do so (typically when N per group is less than 10)
- For each experiment, you should identify the statistical tests used, exact values of N, definitions of center, methods of multiple test correction, and dispersion and precision measures (e.g., mean, median, SD, SEM, confidence intervals; and, for the major substantive results, a measure of effect size (e.g., Pearson's r, Cohen's d)
- Report exact p-values wherever possible alongside the summary statistics and 95% confidence intervals. These should be reported for all key questions and not only when the p-value is less than 0.05.

Please outline where this information can be found within the submission (e.g., sections or figure legends), or explain why this information doesn't apply to your submission:

The statistical analysis methods applied to assess the data from the Autobiographical interview and output from 3-D whole-hippocampal subfield segmentation are described in a subsection at the end of Materials and Methods entitled, Statistical analyses.
Statistical analysis methods applied to conduct whole-brain voxel-based morphometry are described in subsections of the Results, Materials and Methods and in the legend associated with Figure 3. Likewise, statistical analysis methods applied to conducted whole-brain resting-stating fMRI analyses are described in the appropriate subsections Results and Materials and Methods and in the legends associated with Figures 7 and 8.
The type of statistical tests, multiple test correction, and N are identified in the appropriate subsections of the Materials and Methods and Results, and are stated in the Figure Legends.
Study N values are stated in Results, Materials and Methods, and in the Figure Legends.
Exact p-values are reported wherever possible alongside the summary statistics in the Results and Figure Legends for all key questions.
Renders included in Figure 7 and Figure 8 are depicted at the same threshold as used to assess significance (i.e., FDR-corrected $p<0.05$ (two-sided)).

eLife Sciences Publications, Ltd is a limited liability non-profit non-stock corporation incorporated in the State of Delaware, USA, with company number 5030732, and is registered in the UK with company number FC030576 and branch number BR015634 at the address 1st Floor, 24 Hills Road, Cambridge CB2 1JP | August 2014

(For large datasets, or papers with a very large number of statistical tests, you may upload a single table file with tests, Ns, etc., with reference to sections in the manuscript.)

Group allocation

- Indicate how samples were allocated into experimental groups (in the case of clinical studies, please specify allocation to treatment method); if randomization was used, please also state if restricted randomization was applied
- Indicate if masking was used during group allocation, data collection and/or data analysis

Please outline where this information can be found within the submission (e.g., sections or figure legends), or explain why this information doesn't apply to your submission:

No treatment protocols were included as part of experimental design that required random group allocation. Information about the criteria for group allocation (i.e., disease status) are provided in Material and Methods (subsection: Participants)
Subject identity and group membership were masked when scoring the Autobiographical Interview (Materials and Methods, Behaviour, Scoring and Reliability**)**. Likewise, raters were blinded to the identity and group membership of all scans when conducting 3-D quantitative hippocampal subfield morphometry (Material and Methods, Hippocampal subfield quantitative morphometry).
Quantitative metrics were extracted automatically from raw image files for the voxel-based morphometry (subsection in the Result entitled, Whole-brain voxel-based morphometry) and graph theoretic analyses of resting-state data (Fig. 7; Fig. 8) conducted in both the amnesic group and the control group. Identical pre-processing and processing pipelines was applied to both groups.

Additional data files ("source data")

- We encourage you to upload relevant additional data files, such as numerical data that are represented as a graph in a figure, or as a summary table
- Where provided, these should be in the most useful format, and they can be uploaded as "Source data" files linked to a main figure or table
- Include model definition files including the full list of parameters used
- Include code used for data analysis (e.g., R, MatLab)
- Avoid stating that data files are "available upon request"

Please indicate the figures or tables for which source data files have been provided:

Source data for Figure 6 is Table S1
